# A Phylogenetic Analysis of the Genus *Fragaria* (Strawberry) Using Intron-Containing Sequence from the *ADH*-1 Gene

**DOI:** 10.1371/journal.pone.0102237

**Published:** 2014-07-31

**Authors:** Laura M. DiMeglio, Günter Staudt, Hongrun Yu, Thomas M. Davis

**Affiliations:** 1 Department of Molecular, Cellular, and Biomedical Sciences, University of New Hampshire, Durham, New Hampshire, United States of America; 2 Merzhausen, Germany; 3 Department of Medicine, Loma Linda University, Loma Linda, California, United States of America; 4 Department of Biological Sciences, University of New Hampshire, Durham, New Hampshire, United States of America; Washington State University, United States of America

## Abstract

The genus *Fragaria* encompasses species at ploidy levels ranging from diploid to decaploid. The cultivated strawberry, *Fragaria*×*ananassa*, and its two immediate progenitors, *F. chiloensis* and *F. virginiana*, are octoploids. To elucidate the ancestries of these octoploid species, we performed a phylogenetic analysis using intron-containing sequences of the nuclear *ADH-*1 gene from 39 germplasm accessions representing nineteen *Fragaria* species and one outgroup species, *Dasiphora fruticosa*. All trees from Maximum Parsimony and Maximum Likelihood analyses showed two major clades, Clade A and Clade B. Each of the sampled octoploids contributed alleles to both major clades. All octoploid-derived alleles in Clade A clustered with alleles of diploid *F. vesca,* with the exception of one octoploid allele that clustered with the alleles of diploid *F. mandshurica*. All octoploid-derived alleles in clade B clustered with the alleles of only one diploid species, *F. iinumae*. When gaps encoded as binary characters were included in the Maximum Parsimony analysis, tree resolution was improved with the addition of six nodes, and the bootstrap support was generally higher, rising above the 50% threshold for an additional nine branches. These results, coupled with the congruence of the sequence data and the coded gap data, validate and encourage the employment of sequence sets containing gaps for phylogenetic analysis. Our phylogenetic conclusions, based upon sequence data from the *ADH*-1 gene located on *F. vesca* linkage group II, complement and generally agree with those obtained from analyses of protein-encoding genes *GBSSI*-2 and *DHAR* located on *F. vesca* linkage groups V and VII, respectively, but differ from a previous study that utilized rDNA sequences and did not detect the ancestral role of *F. iinumae*.

## Introduction

The genus *Fragaria* (strawberry) belongs to the economically important Rosaceae family, subfamily Rosoideae. The modern cultivated strawberry is an important fruit crop that is grown in over 60 countries, and that in 2012 had a worldwide production of over 4.5 million metric tons [1] and a total crop (fresh market and processing fruit) value of over $2.4 billion in the United States, up from $1.7 billion in 2007 [1]. Like many important crop species, such as bread wheat (*Triticum aestivum*) and cotton (*Gossypium hirsutum*), the history of the cultivated strawberry involves both hybridization and polyploidization. Yet current understanding of the cultivated strawberry’s complex octoploid (2n = 8x = 56) genome composition and reticulate evolutionary history is limited, and relies on just a few molecular studies [2], [3], [4], [5], [6]. Herein, we report the use of a highly variable intron sequence from a nuclear, protein-encoding gene, alcohol dehydrogenase 1 (*ADH-*1) to study molecular diversity and phylogenetic relationships within *Fragaria* with the aim of tracing the cultivated strawberry’s ancestry to the diploid level.

The octoploid, cultivated strawberry, *Fragaria*×*ananassa* originated in Europe in the mid-1700s from hybridization between the ancestral octoploids *Fragaria chiloensis* and *Fragaria virginiana* [7]. These octoploids are native to South and North America, respectively, but had been brought to Europe and were being grown in proximity in European horticultural gardens [8], where hybridization ensued. The hybrids were easily recognizable by their distinctive and generally desirable characteristics, including their sizable, fragrant, and pale red fruit and their exceptional vigor [8], on which basis they were brought into cultivation and breeding [7]. Thus, the immediate and very recent ancestry of the cultivated strawberry is evident as a matter of historical record, and to further trace its ancestry is to explore the origin(s) of its octoploid progenitors.

The genus *Fragaria* is currently considered to encompass about 24 species [9], [10], which have been described on the basis of morphological features, geographic distribution, ploidy [7], [11], cross-fertility, and known hybridity. The basic chromosome number is *x* = 7 [12], and the *Fragaria* species with even, euploid chromosome numbers form a polyploid series that includes diploid (2*n* = 2*x* = 14), tetraploid (2*n* = 4*x* = 28), hexaploid (2*n* = 6*x* = 42), octoploid (2*n* = 8*x* = 56), and decaploid (2*n* = 10*x* = 70) members. On the basis of botanical evidence, tetraploids *F. orientalis* and *F. tibetica* may be derived, respectively, from diploids *F. mandshurica* [13] and *F. pentaphylla* [14], while tetraploid *F. moupinensis* may be derived from diploid *F. nubicola*, and tetraploids *F. gracilis* and *F. corymbosa* may both be derived from an as-yet-undescribed diploid species [10].

Many interspecific hybrids have been described in *Fragaria*, among which triploids (3*x*), pentaploids (5*x*), and various other “odd-ploids” are represented [15], [16]. Ploidies vary within each of two hybrid species, *F*. ×*bringhurstii* and *F*. ×*bifera* [17], [18]. The newly described decaploid *F. cascadensis* may be a hybrid derivative of octoploid *F. virginiana* subsp. *platypetala* and *F. vesca* subsp. *bracteata* [19]. Interspecific hybridizations are also thought to have played a role in the origins of hexaploid *F. moschata* and the two ancestral octoploid *Fragaria* species [7], implying that these higher level polyploids have allo- or alloauto- polyploid genome compositions.

Chromosomes are uniformly small in all *Fragaria* species [12], [20], and chromosome morphology has provided no phylogenetic illumination. Based upon results of meiotic pairing studies in various hybrids, three octoploid genome composition models have been proposed: Model I - AAAABBCC [21]; Model II - AAA’A’BBBB [20]; and Model III - AAA’A’BBB’B’ [22]. Model I implies the existence of three distinct subgenome types (A, B, and C), each presumably derived from a different diploid ancestor. In contrast, models II and III postulate just two well-differentiated subgenome types: A-type and B-type, with lesser degrees of differentiation occurring within one (Model II: A versus A’) or both (Model III: A versus A’ and B versus B’) of these two major types. Based upon molecular phylogenetic evidence, Rousseau-Gueutin et al. [4] have proposed two alternate models: Y1’Y1’Y1’’Y1’’ZZZZ (equivalent to Model II) and Y1Y1Y1Y1ZZZZ. Because each distinct model postulates a unique pattern of octoploid subgenome representation and differentiation, each model implies a different phylogenetic hypothesis with respect to the numbers of ancestral diploids and their respective degrees and patterns of differentiation.

*Fragaria* has been represented in four molecular phylogenetic studies of the Rosaceae family [23], [24], [25], [26]. However, each of these broad studies included only one or two *Fragaria* species: (*F.* ×*ananassa* [23], *F. vesca* and *F.* ×*ananassa* [24], [25], or *F. vesca* and *F. virginiana* [26]); and provided no insight into species relationships within *Fragaria*. In a recent study [27] of the Rosoideae subclade Fragariinae that included one representative for each of six *Fragaria* species (diploids *F. vesca* and *F. viridis*, hexaploid *F. moschata*, and octoploids *F. chiloensis*, *F. virginiana*, and *F.* ×*ananassa*), analysis of plastid sequences found that the octoploids had a closer phylogenetic affinity to *F. vesca* than to *F. viridis*. Overall, the monophyly of *Fragaria* is well supported [3], [27]. Eriksson et al. [24], [26] found *Fragaria* to be a subclade within paraphyletic *Dasiphora*, wherein *D. fruticosa* was one of *Fragaria*’s closest sisters [26]. Subsequently, Lundberg et al. [27] depicted a sister relationship between *Fragaria* and a clade containing the genera *Dasiphora*, *Drymocallis*, *Chamaerhodos*, and *Potaninia*. Hence, *Dasiphora fruticosa* (formerly *Potentilla fruticosa*) was justifiably used as the outgroup in our study, as it was also used by Potter et al. [3].

Harrison et al. [2] used chloroplast DNA (cpDNA) restriction fragment length polymorphisms (RFLP) in the first molecular study of relationships within the genus *Fragaria*, and found a close affinity between representatives of *F. virginiana* and *F. chiloensis,* suggesting that these two species may have arisen from a common octoploid ancestor. Subsequently, Potter et al. [3] utilized nuclear internal transcribed spacer (ITS) and cpDNA *trn*L-*trn*F sequence data to study relationships within *Fragaria*. The cpDNA sequence analysis was sufficient to define a multi-species clade A consisting of the octoploid species and two diploids: *F. vesca* and “*F. nubicola”* [3]. Here, it must be noted that the “*F. nubicola”* accession (CFRA 520) studied by Potter et al. [3] has been re-identified as *F. bucharica* [9], [28], and thus it is *F. bucharica* and not *F. nubicola* that was shown to have phylogenetic affinity to *F. vesca* and the octoploids. ITS data [3] defined a clade comprised of the aforementioned species but also including *F. orientalis* (4*x*) and *F. moschata* (6*x*). Analysis of the combined cpDNA and ITS data sets also defined a modestly supported clade B comprised of Asian species *F. nipponica*, *F. gracilis*, *F. pentaphylla*, *F. daltoniana* and *F. nilgerrensis*. Notably, diploid *F. iinumae* was considered the most divergent *Fragaria* species based on cpDNA RFLP analysis [2], and as sister to all other *Fragaria* species in the combined ITS/cpDNA sequence-based analysis [3].

Although cpDNA and ITS sequences are commonly used for phylogenetic resolution at the species level, neither is likely to reveal the reticulate phylogenetic history of allopolyploid species. While the cpDNA and ITS studies of Potter et al. [3] drew attention to *F. vesca*, *F. bucharica*, and *F. orientalis* as possible progenitors to the octoploids, neither study discerned the reticulate phylogenetic history expected for the octoploids or for hexaploid *F. moschata*. Of course, uniparentally inherited cpDNA sequence alone cannot provide evidence of phylogenetic reticulation. In order for reticulation to be revealed, alleles from both contributing diploid genomes must be retained in a polyploid and detected in its analysis. Nuclear ITS sequences are bi-parentally inherited, but are subject to concerted evolution, which could potentially erase evidence of one or the other contributing diploid allele in an advanced allopolyploid lineage [29]. In allotetraploid cotton, concerted evolution has homogenized the ITS regions both within and between contributing genomes, effectively erasing the original ITS contribution of all but one of the original diploid ancestors [29]. While no direct evidence exists that concerted evolution has occurred in the ITS region of *Fragaria*, Potter et al. [3] only detected single ITS forms in the hexaploid and octoploid *Fragaria* species, indicating that ITS loss through homogenization could have been a factor in *Fragaria* genome evolution. Alternately, ITS loss *per se* may provide an explanation: fluorescent in situ hybridization using ribosomal DNA (rDNA) probes has suggested possible evolutionary elimination of rDNA sites in the octoploid *Fragaria* species [30].

In an effort to avoid the problems associated with concerted evolution and uniparental inheritance, we chose to employ intron-containing sequence from the nuclear alcohol dehydrogenase gene, *ADH-*1, to study phylogenetic relationships in *Fragaria*. The *ADH* gene is among the most widely studied plant genes, existing as a small gene family in most plants [31], but as a single copy gene in *Arabidopsis* [32]. *ADH* sequence comparisons were found to be highly informative for phylogenetic studies in *Paeonia*, [33] and *Gossypium* [34], two genera in which hybrid speciation and/or allopolyploidy have had significant roles. Notably, an *ADH* gene was the first protein-encoding gene to be completely sequenced in strawberry [35], and this sequence was the basis for design of the PCR primer pair used in our research. Using these primers, we amplified and mapped the *ADH* locus to *F. vesca* linkage group II [36]. Subsequently, sequencing of a genomic DNA (fosmid) clone of *F. vesca* subsp. *americana* ‘Pawtuckaway’ (GenBank accession number EU024832) revealed that a pair of adjacent *ADH* genes exists at the respective locus [37]. Of these two genes, the one most closely resembling the original *ADH* sequence of Wolyn and Jelenkovic [35] was designated *ADH*-1, and the other as *ADH*-2 [37]. The present study is concerned with sequence variation in *ADH*-1, to which the employed primer pair is specific.

Similarly, Rousseau-Gueutin et al. [4] explored the phylogenetic utility of two nuclear, protein-encoding genes: granule-bound starch synthase I-2 (*GBSSI*-2 = *Waxy*) and dehydroascorbate reductase (*DHAR*). These genes are determinable by Blastn search of the *Fragaria vesca* reference genome (Strawberry Genome v1.1 pseudomolecules), which is archived on the Genome Database for Rosaceae website (http://www.rosaceae.org/node/1), to reside on linkage groups V and VII, respectively. Their results [4] provided support for allopolyploid origins of hexaploid *F. moschata*, octoploids *F. chiloensis* and *F. virginiana*, and *F. iturupensis* – the latter taxon comprising accessions that have been variously described as octoploid [38] and decaploid [39].

Although protein-encoding genes may also be subject to interlocus concerted evolution, gene families with low copy numbers are less susceptible than are high copy number families [34]. In addition, rapidly evolving intron sequences may be particularly phylogenetically informative at high levels of relationships where nucleotide variability in coding regions is rare [40], [41]. Nuclear intron sequences have been used to illuminate phylogenetic relationships in both plant and animal species [42], [43], [44].

One potential problem with using intron sequences for phylogenetic reconstruction is that they often contain insertion/deletion (indel) polymorphisms that require the introduction of gaps into multiple sequence alignments. Gaps are typically assumed to introduce ambiguity to multiple alignments, and regions that contain them are often treated as missing data in phylogenetic analyses [45], [46]. This trend has been challenged recently with the accumulation of an increasing amount of evidence suggesting that indels contain a phylogenetic signal that should not be ignored [45], [46]. We have explored the phylogenetic utility of indel polymorphisms in this analysis.

## Materials and Methods

### Plant Materials

A total of 38 *Fragaria* accessions representing 19 species (Tables 1, S1), and one representative of outgroup species *Dasiphora fruticosa* were included in this analysis. *Fragaria* germplasm accessions were obtained from four sources: USDA National Clonal Germplasm Repository (NCGR), Corvallis, Oregon [accessions with CFRA prefixes and USDA Plant Introduction (PI) numbers]; the collection of Günter Staudt in Merzhausen, Germany (accessions with ST prefixes); our own collection (GS2C, PAWT, and U2A), and W. Atlee Burpee and Co., Warminster, PA (‘Yellow Wonder’ = YW). The wild accessions were collected in the geographic regions listed in Table 1; with more precise locations, where available, provided in Table S1.

**Table 1:** Species represented in the phylogenetic study, and their continental origins.

SPECIES	ACCESSIONS	CONTINENTAL ORIGINS
**Diploids**		
*F. bucharica* Losinsk.	CFRA 520	Central Asia (Pakistan)
	ST 4952	Central Asia (Pakistan)
	ST 93, 2-1	Central Asia (Pakistan)
*F. daltoniana* J. Gay	ST 4949	Central Asia (Nepal)
*F. nubicola* (Hook. f.) Lindl, ex Lacaita	ST 96, 4-1	Central Asia
*F. iinumae* Makino	CFRA *377*	Eastern Asia (Japan)
	CFRA 1849	Eastern Asia (Japan)
	CFRA 1853	Eastern Asia (Japan)
	ST 95,6	Eastern Asia (Japan)
*F. mandshurica* Staudt	ST 99, 2-4	Northeastern Asia
	ST 20, 1-3	Northeastern Asia
*F. nilgerrensis* Schltdl. Ex J. Gay	CFRA 1223	Asia (China)
*F. vesca* L. subsp. *vesca*	‘Yellow Wonder’ (YW)	Europe
	ST 97, 6	Europe
	ST 95, 9	Europe
	CFRA 282	Europe
	ST 4959	Europe
*F. vesca* subsp. *americana* (Porter) Staudt	‘Pawtuckaway’ (PAWT)	Eastern North America
*F. vesca* subsp. *bracteata* (Heller) Staudt^1^	U2A	Western North America
	BC21	Western North America
*F. viridis* Weston	CFRA 341	Europe
	ST 4942	Asia (Russia)
*F. nipponica* Makino	ST 96, 7-4	Eastern Asia (Japan)
	CFRA 1009	Eastern Asia (Japan)
*F. pentaphylla* Losinsk.	ST 92, 1-5	Central Asia (China)
	ST 4973	Asia (China)
**Tetraploids**		
*F. corymbosa* Losinsk.	ST 96, 18-3	Eastern Asia (China)
*F. gracilis* Losinsk.	ST 97, 7-2	Asia (China)
*F. moupinensis* (Franch.) Cardot	ST 3988	Asia
*F. orientalis* Losinsk.	ST 95	Asia
*F. tibetica* Staudt & Dickoré	ST 99, 1-5	Asia (China)
	ST 4972	
**Hexaploid**		
*F. moschata* Weston	CFRA 157	Europe
**Octoploids**		
*F. chiloensis* (L.) Mill. subsp. *lucida* (E. Vilm. Ex Gay) Staudt	CFRA 366	Western North America
*F. chiloensis* (L.) Mill. subsp. *chiloensis* f. *chiloensis*	CFRA 742	South America (Chile)
*F. virginiana* Mill. subsp. *virginiana*	CFRA 67	Eastern North America
*F. virginiana* Mill. subsp. *glauca* (S. Watson) Staudt	CFRA 370	Western North America
**Decaploid**		
*F. iturupensis* Staudt	CFRA 1841	Western Asia
**Outgroup**		
*D. fruticosa* (L.) Rydb.	D. fruticosa	North America

Further details about the studied germplasm accessions are provided in Supplementary Table S1.

1. The accessions listed as *F. vesca* ssp. *bracteata* were collected within the range of this subspecies, as delineated by Staudt (1999), but have not been definitively typed.

Notably, our investigation included a widely studied and utilized accession, CFRA 520 ( = PI551851 = IPK accession 94056-33.K = FDP 601), that had initially been misidentified as *F. nubicola* [28] and referred to as *F. nubicola* by a host of researchers [2], [3], [47], [48], [49], [50], [51], [52], [53], [54], [55]. This error was recognized and corrected by Staudt [28], who reclassified CFRA 520 as *F. bucharica*.

### DNA Amplification and Sequencing

Genomic DNA was extracted from young, partially expanded leaves using a standard CTAB miniprep protocol patterned after Torres et al., [56]. PCR primers ADH2F (5′-ccaaggtacacattctttttttc-3′) and ADH3R (5′-GTCACCCCTTCACCAACACTCTC-3′) were designed on the basis of published *ADH-*1 genomic sequence from *F*. ×*ananassa* [35] to specifically target a region spanning intron 2, exon 3 and intron 3 of the *ADH-*1 gene. The target site of primer ADH2F extends from the end of exon 2 seventeen bases into intron 2, while that of primer ADH3R is entirely within exon 4. PCR amplifications were performed in 25 µl reactions using Eppendorf reagents (1X buffer solution, 1 unit *Taq* polymerase, 2.0 mM total Mg(OAc)_2_, 1X TaqMaster), 100 µM each dNTP, 0.4 µM each primer, and 100 ng template DNA. The PCR protocol consisted of thirty cycles. Each step was one minute long, and 94°C denaturation, 58°C annealing, and 72°C extension temperatures were utilized. Products were visualized on 2% agarose TBE gels stained (post-electrophoresis) with ethidium bromide.

PCR products were cloned into the TOPO TA vector (Invitrogen) from all accessions prior to sequencing. Colonies were screened using the PCR protocol listed above, and either the M13F and M13R vector primers or the ADH2F and ADH3R specific primers were employed, using a small amount of the cloned colony as the template DNA. For each diploid and tetraploid accession, ten colonies were subjected to PCR screening, and if a length polymorphism was detected, one clone of each electrophoretic band mobility variant was chosen at random and sequenced. If no length variation was detected within an accession, a single clone was chosen at random to be sequenced. For each hexaploid and octoploid accession, at least 30 colonies were screened, and at least ten colonies per accession were sequenced, with care taken to ensure that all evident band mobility variants within each accession were represented by the chosen colonies. Overnight subcultures of selected clones were grown in 3 ml liquid LB media with 50 µg/ml ampicillin. Plasmids were then purified using Wizard Minipreps (Promega). Sequencing reactions were performed in both directions using the M13F and M13R vector primers and utilized Amersham DYEnamic ET terminator cycle sequencing chemistry. Sequencing gels were run on an ABI 377 sequencer (Applied Biosystems).

### Assessing ADH gene copy number

A Southern hybridization was conducted to verify that the amplified region targeted for sequencing is single copy in diploid *Fragaria* species. Three different restriction enzymes were used at a concentration of 2 units per µg of DNA to cut 6 µg genomic DNA from FRA1223 (*F. nilgerrensis*), FRA377 (*F. iinumae*), YW and U2A (both *F. vesca*, but different subspecies). Reaction products were separated by gel electrophoresis on a 0.8% agarose gel and stained with ethidium bromide. Fragments were transferred to an Immobilon Ny+ membrane according to manufacturer’s instructions (Millipore).

The α-p32 radiolabeled probe was generated via PCR according to Sambrook and Russell [57] from plasmid DNA containing the *ADH*-1 target region acquired from the *F. vesca* accession PAWT. Prehybridization and hybridization were performed in 5X SSPE, 5X Denhardts, 1% SDS, and 100 µg/ml salmon sperm DNA at 64°C with a two hour prehybridization and 15 hour hybridization. Membranes were then washed twice at room temperature for five minutes each (2X SSC, 0.1% SDS) and twice at 64°C for 15 minutes each (0.2X SSC, 0.1% SDS). Membranes were exposed for 48 hours.

### Sequence Alignment and Phylogenetic Analyses

Sequences were edited using SeqEd ver 1.08 (Applied Biosystems), and components of the LaserGene suite of programs (DNASTAR), including MegAlign and EditSeq, were used for various associated purposes. Preliminary sequence alignments were used to identify and eliminate sequence redundancy within accessions: a sequence was considered redundant if identical to or differing from another allele within that accession by only a single, autapomorphic base substitution. ClustalW ver. 1.83 [58] was used for the multiple sequence alignment using the default settings and manual “by eye” adjustments. Since the alleles in our data set contained several length polymorphisms, our final alignment contained multiple gaps. Parsimony informative gaps were then coded as binary characters according to the “simple indel coding” method of Simmons and Ochoterena [59]. The binary gap data and the sequence data were treated as separate partitions.

All phylogenetic tests were conducted in PAUP 4.0b10 [60] unless otherwise stated. A 1000 replicate permutation tail probability (PTP) test was performed on the nucleotide data to test for phylogenetic signal. The heuristic search consisted of 10 random starting tree searches per replicate, withTBR branch swapping, and MULtrees option in effect. Each replicate was limited to 3×10^7^ rearrangements. A 100 replicate partition homogeneity test was performed on the unweighted data set to test for congruence between the nucleotide partition and the gap partition.

Unweighted maximum parsimony (MP) analyses were conducted on the sequence data alone, and also on the sequence data with the coded binary gap characters added to the end of the data matrix. All characters were unordered and aligned gaps were treated as missing data, whether or not coded gap characters were included in the analysis. The default parsimony settings were used and heuristic searches consisted of 1000 replicates using random starting trees with TBR branch swapping, and MULtrees in effect. Strict consensus trees were obtained from all most-parsimonious trees in each analysis. Bootstrap analyses [61] were conducted using 10,000 “fast” stepwise addition pseudoreplicates.

Differentially weighted MP analyses were also conducted on both data sets (with and without binary gap characters) using the above search settings. Maximum Likelihood (ML) was used to estimate the transition to transversion ratio (ti/tv) using a randomly chosen unweighted MP tree. The ti/tv ratio was then used in a two-step matrix to differentially weight transversions. When coded gaps were included in the weighted analysis, they were given the same weight as transversions. Bootstrap analyses were conducted as for the unweighted analyses.

The most appropriate evolutionary model to use in the Maximum Likelihood analysis was determined by the Bayesian inference criterion (BIC) and the corrected Akaike Information Criterion (AICc) using the program JModeltest 0.1.1 [62], [63]. The analyses resulted in different best-fit evolutionary models, and a Maximum Likelihood (ML) analysis was performed for each model. ML analyses were performed using 20 random addition heuristic searches starting from a stepwise addition tree with TBR branch swapping. Bootstrap analyses consisted of 10,000 “fast” stepwise addition pseudoreplicates.

## Results

### Sequence variation

Using the described sampling strategy, one or more *ADH-*1 alleles were obtained from each accession. For all *Fragaria* accessions, sequence length varied over a range of 446 bp to 639 bp (Table S2). Among diploid accessions, most had a single detected allele. However, two alleles were differentiated in each of three diploid accessions: *F. bucharica* accession CFRA 520 and *F. mandshurica* accessions ST 99,2–4 and ST 20,1–3 (Table S2). A single allele was detected in each of the five tetraploid accessions examined, while three allele variants were detected in the *F. moschata* (hexaploid) accession, and four to eight allele variants were detected in each of the octoploid accessions. Two problematic octoploid-derived alleles were identified as probable PCR recombinants and were excluded from further consideration. *D. fruticosa* had an allele size of only 323 bp due to deletions in intron 2.

**Figure 1:**
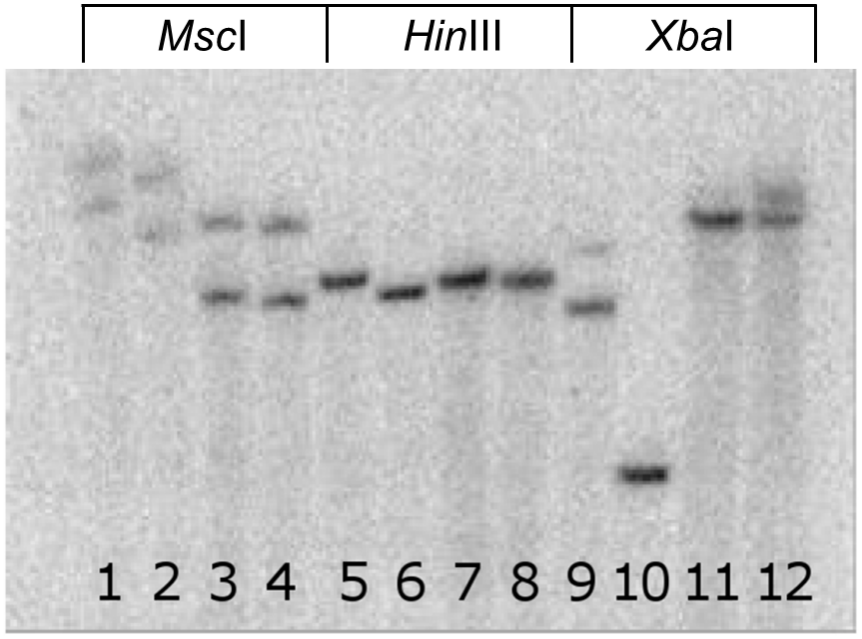
Genomic Southern blot. Autoradiograph of genomic southern blot using a P32 labelled probe of *ADH*-1 target region from *F. vesca* accession PAWT. In lanes 1–4, genomic DNA from FRA1223 (*F. nilgerrensis*), FRA377 (*F. iinumae*), U2A (*F. vesca*) and YW (*F. vesca*), respectively, were digested with *Msc*I, which has one expected cut site in the target DNA. In lanes 5–8 the above accessions were digested with *Hin*dIII and those in lanes 9–12 were digested with *Xba*I, both with no expected cut sites in the target DNA.

An examination of *ADH-*1 copy number by Southern hybridization in four diploid accessions representing three species indicated that the sequenced region was present in a single copy in these accessions. Based upon the obtained sequence data, the restriction enzymes *Hin*dIII and *Xba*I had no expected cut sites within the target sequence, while *Msc*I was expected to cut once. On the resulting genomic Southern (Figure 1), all four accessions yielded single electrophoretic bands when digested with *Hin*dIII, and a pair of bands when cut with *Msc*I, the respective results being as expected for a single copy target sequence lacking or containing a single cut site. The *Xba*I digests yielded one bright band in each species, but a light, fuzzy band was also present in the lanes containing FRA1223 and YW (Figure 1). This apparent inconsistency with results from the other two enzymes may be due to incomplete digestion of the genomic samples by *Xba*I.

### Sequence Alignment and Phylogenetic Analyses

A total of 72 sequences generated from 38 *Fragaria* germplasm accessions, and one sequence from outgroup species *Dasiphora fruticosa*, were included in the phylogenetic analysis. The sequences have been deposited into GenBank under accession numbers KJ606694–KJ606765, as listed in Table S2. Since the data set encompassed alleles of different read lengths, multiple gaps were necessarily introduced into the final alignment. Placement of gaps was unambiguous with the exception of one region of five nucleotides in intron 2. For this region, we chose the alignment that required the fewest substitutions. The final alignment of 72 sequences had a length of 663 nucleotide characters, excluding primer sites (Table S2). The simple indel coding method [59] resulted in 26 binary characters being appended to the alignment (Table S2) for a total length of 689 characters. All but one of these gaps occurred in intron 2, with the exception located in intron 3. There were 70 parsimony informative characters in the sequence data alone and 96 parsimony informative characters when gaps were included (all coded gaps were parsimony informative). The PTP test indicated that our data set provides a significantly better than random phylogenetic signal (P = 0.001), and the partition test did not indicate incongruence between the DNA sequence and gap partitions (P = 0.41).

**Figure 2:**
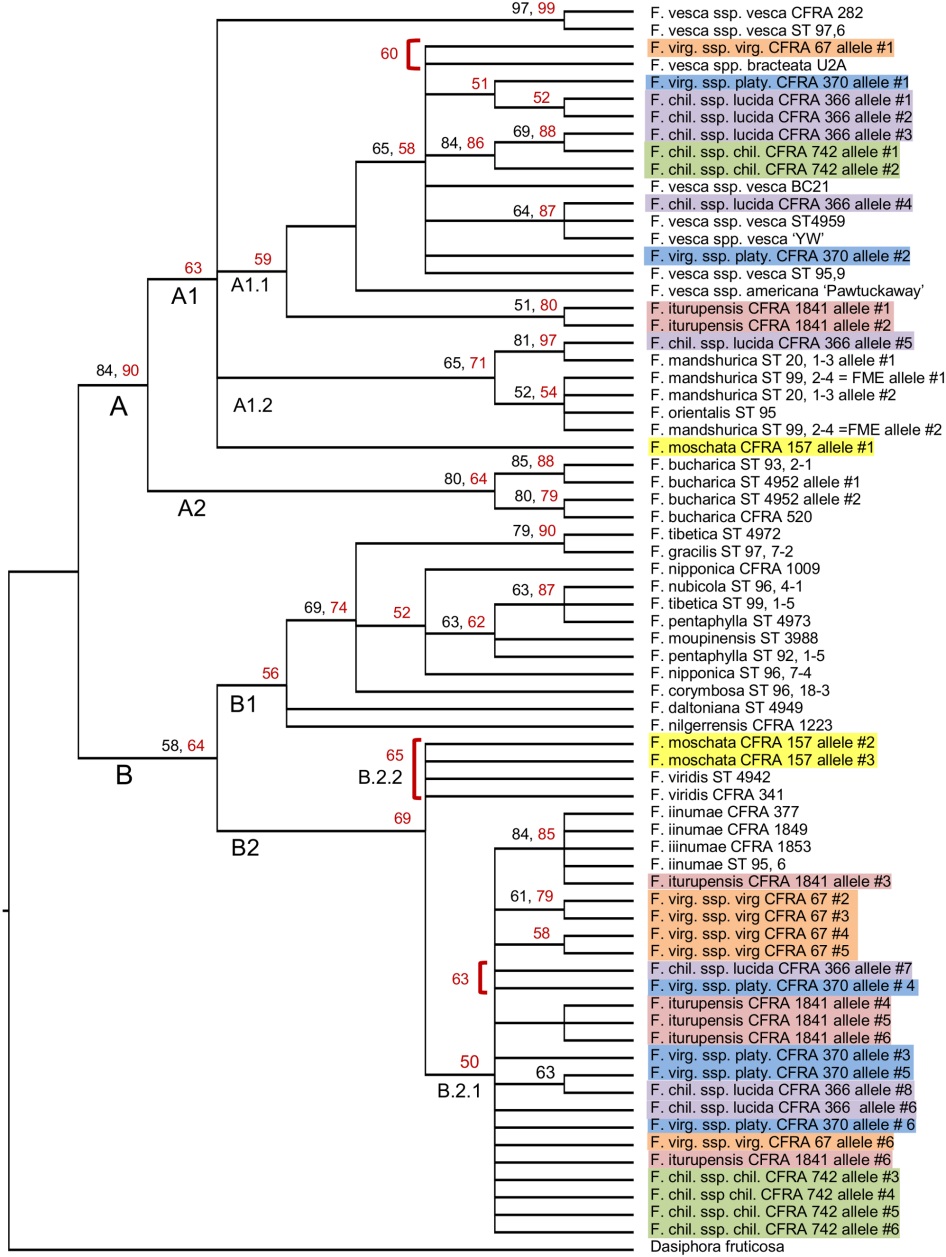
Strict consensus phylogenetic tree from MP analyses excluding and including gap characters. The depicted tree structure resulted from an MP analysis on the unweighted data set with binary coded gap characters excluded. Bootstrap consensus values >50% are shown in black for the analysis conducted with gaps excluded, and in red for that on the unweighted data with coded gap character included. Red brackets define three clades that resolved only when gaps were included in the MP analysis and had bootstrap support >50%. The inclusion of gaps also produced four additional nodes in Clade B.2.1 that did not have bootstrap support over 50% and are not shown in this figure. The polyploid accession names are differentially color-highlighted according to species.

**Figure 3:**
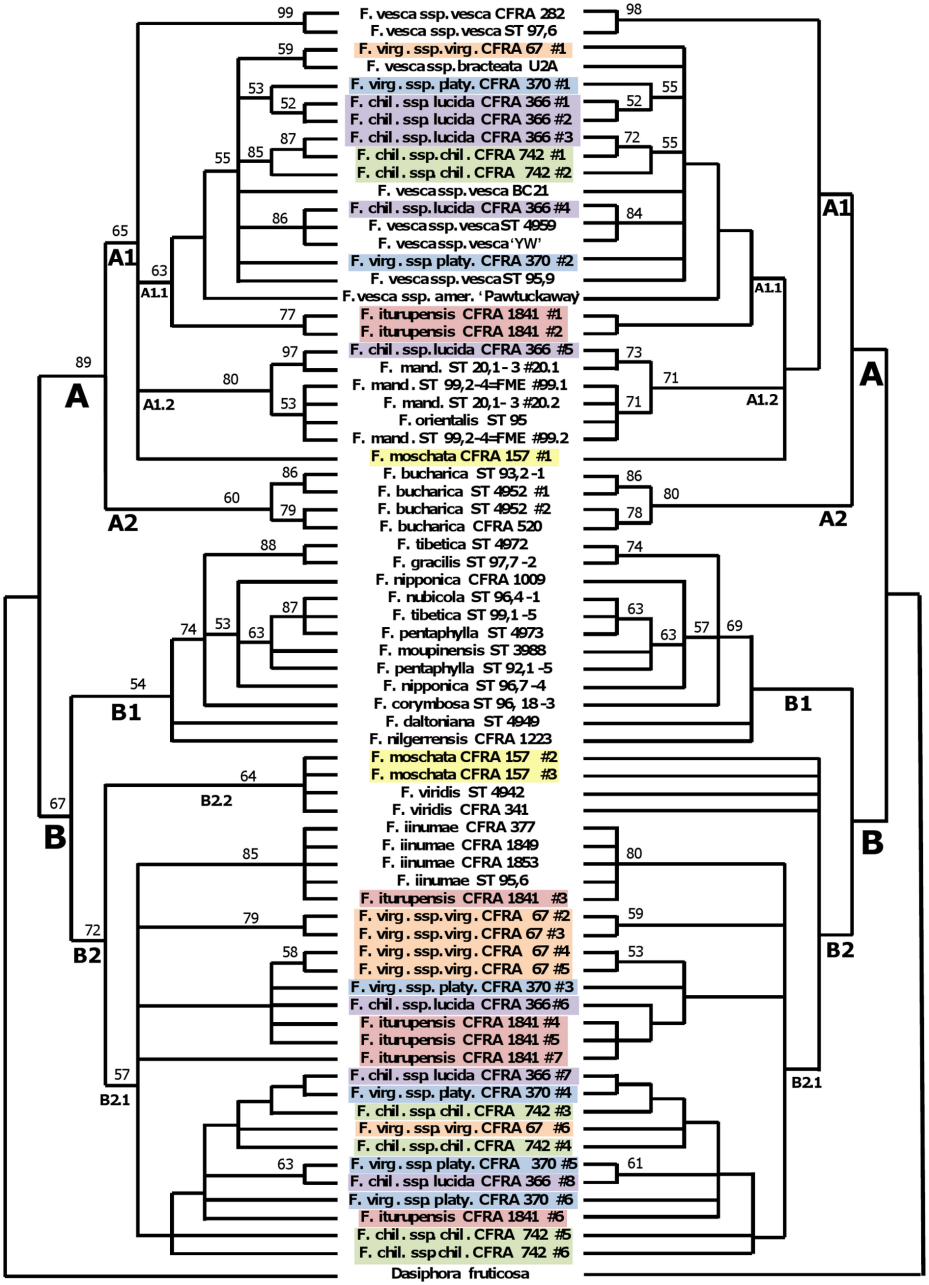
Comparison between most resolved MP and ML trees. The phylogenetic tree on the left was produced using an MP analysis on the data set with binary coded gaps included and both coded gaps and transversions given a weighting of 1.5. The phylogenetic tree on the right was produced using ML and the TPM1uf+G evolutionary model as indicated by JModeltest (Posada, 2008; Guindon Gascuel 2003). Bootstrap support values above 50% are shown on both trees.

The unweighted MP analysis on the sequence data alone produced 400 most parsimonious trees (score = 217), with 33 nodes resolved in the strict consensus tree and 21 branches with bootstrap support over 50% (Figure 2). When transversions are given the weight of the estimated ti/tv ratio of 1.5 and coded gaps are excluded from the analysis, 486 best trees (score = 261.0) were produced, with 33 resolved nodes in the strict consensus tree and 24 branches with bootstrap support over 50% (not shown). Phylogenetic resolution, as indicated by the number of nodes, was enhanced by including our binary coded gaps in the unweighted analysis. The inclusion of gaps into the unweighted data set produced 2343 most parsimonious trees (score = 249) with 39 nodes resolved in the strict consensus tree and 32 branches with bootstrap support over 50%. The respective bootstrap support values are shown in Figure 2. One additional node is in the A1 clade and the rest are in the B2 clade. Five additional branches with bootstrap support above 50% were located in the A clade while six added to the B clade. Our most resolved tree, however, resulted when gaps were included and both gaps and transversions were given the weight of 1.5. This analysis produced 2183 most parsimonious trees (score = 308.5) with 40 nodes resolved in the strict consensus tree and 32 branches with bootstrap support over 50% (Figure 3).

The BIC and the AICc analyses, as implemented in JModeltest [62], [63], produced different best fit evolutionary models. The BIC determined HKY+G to be the best model. This model indicates that base frequencies are unequal, that rates vary among sites, that there are no invariable sites, and has two rate categories, one for transitions and one for transversions. Base frequencies were f(A) 0.3208, f(C) 0.1599, f(G) 0.1689, and f(T) 0.3504. The ti/tv ratio was 1.5047 and the gamma shape was 1.0660 with 4 gamma distribution categories. The AICc analysis determined TPM1uf+G to be the best fit model. This model also indicates that base frequencies are unequal, that rates vary among sites, that there are no invariable sites, but has three rate categories, two for transversions and one for transitions. Base frequencies were 0.3235, 0.1576, 0.1662, and 0.3527 with substitution rates of rAC = 1.000, rAG = rCT = 2.6305, and rAT = rCG = 0.5860 and a gamma shape of 1.0510 with 4 gamma distribution categories.

The Maximum Likelihood (ML) tree produced from the settings described in the AICc analysis is displayed in Figure 3, with 41 resolved nodes and 21 branches with bootstrap support over 50%. The tree based on the BIC settings was identical in structure and is not shown. The ML tree structure is similar to that of the most highly resolved MP trees.

All MP analyses produced strict consensus trees having all *Fragaria* alleles encompassed and similarly distributed in two major clades, designated A and B (as represented by Figures 2 and 3). Both ML analyses defined the same major clades, closely resembling the MP trees. All alleles in clade A had read lengths in the range of 539 to 639 bp, while all those in clade B were in the distinctly smaller range of 446 to 493 bp.

Each major clade included subclades. Subclade A1 encompassed all alleles from *F. vesca*, *F. mandshurica*, and *F. orientalis*, one allele from hexaploid *F. moschata*, and at least one allele from each of the five octoploid accessions. Its sister subclade A2, encompassed the four alleles from *F. bucharica*. Subclade A1.1 was limited to alleles from *F. vesca* and the octoploid species. Subclade A1.2 encompassed all alleles from *F. mandshurica* and *F. orientalis*, and one allele from *F. chiloensis.* Subclade B1 consisted of all alleles from five Asian diploids and four Asian tetraploids. Subclade B2 subdivided into sister subclade B2.1, consisting of all alleles from diploid *F. iinumae* and at least two alleles from each octoploid accession, and subclade B2.2 (not present in ML tree), consisting of all alleles from diploid *F. viridis* and two *F. moschata* alleles. Overall, alleles from the five octoploid accessions fell into either subclade A1 or subclade B2.1, with each octoploid accession having allele representation in both of these subclades. Alleles from the *F. moschata* accession fell into subclades A1 and B2.2.

## Discussion

The sequenced region of the *Fragaria ADH-*1 gene proved to be a rich source of informative nucleotide substitutions and indel polymorphisms. Use of the ADH2F-ADH3R primer pair, in which the ADH2F primer had been designed to extend well into intron 2 to help assure target specificity, generated no PCR product sequences that could not be easily entered into the alignment. The corresponding region of the adjacent gene copy, *ADH-*2, is markedly different from *ADH-*1 in exon and particularly intron nucleotide sequence [37], precluding confusion between the two genes, and no sequences resembling *ADH-*2 were recovered from the cloned PCR products. The results of the genomic Southern blot indicated that only a single copy of the target sequence existed in the genomes of four diploid accessions representing three species and two *F. vesca* subspecies. The only species that have multiple alleles distributed to differing major clades were the hexaploid and octoploid species, in which allopolyploid genome constitution would prompt anticipation of just such an allele distribution. In total, these results strongly support the interpretation that no paralogy exists in the data set except that arising from allopolyploid duplication of a common ancestral ortholog.

Our sampling strategy sought to minimize the number of sequences generated from each accession, but in doing so did not appear to sacrifice phylogenetic information. Each of the diploid accessions is represented by a single allele sequence in this study, with the exception of one *F. bucharica* and two *F. mandshurica* accessions. In each of these three recognizably heterozygous accessions, two allele types were initially differentiated on the basis of electrophoretic band mobility polymorphisms, upon which criterion two alleles were sequenced in these species. Heterozygosity is not surprising in *F. bucharica* and *F. mandshurica*, which have gametophytic self-incompatibility systems [11]. The only effect of sampling alternate alleles in these three heterozygous diploid accessions was the additional ramification of three minor, terminal clades, adding no nodes to the trees and having no effect on species-level phylogenetic resolution. None of the five studied tetraploid species were considered by Staudt [10] to have arisen via interspecific hybridization [10], and no instance of allotetraploidy in *Fragaria* was resolved by Rousseau-Gueutin et al. [4]. Given the wide diversity of sequence read lengths and concomitant electrophoretic band mobility variation among the diploid-derived alleles, it is likely that an allotetraploid genomic constitution would have resulted in detectable band mobility variation within an allotetraploid accession, if present, but none were detected. This observation suggests that additional allele sampling within the studied diploid and tetraploid accessions would not have added material resolution to the analysis. However, broader sampling of tetraploid germplasm would be desirable to assess the consistency of allele and genome constitution within each of the tetraploid species.

At the octoploid level, our objective was not necessarily to capture every allelic variant within an accession, but to capture as many phylogenetically informative variants as possible; hence, care was taken to sequence all detectable electrophoretic mobility variants, although multiple clones within the same electrophoretic mobility class were also sequenced, providing opportunity to detect additional variation unrelated to read length. Given that the read lengths of alleles in the major clades A and B were distributed in non-overlapping ranges, our sampling strategy had the effect of assuring that alleles belonging to both major clades would be detected, if present, in each octoploid accession.

In the MP phylogenetic analyses, resolution was increased considerably by including coded gap characters. The number of nodes increased from 33 to 39 and the number of branches with bootstrap support >50% increased from 21 to 32. Weighting of transversions alone did not produce any more nodes than did the unweighted analysis without gap characters, but weighting of transversions did increase the number of branches with bootstrap support >50% from 21 to 24. The most resolved tree resulted when gaps and transversions were both employed and weighted at the same elevated level; however the improvement was marginal as compared with the MP treatment that included unweighted, coded gap characters. The addition of weight to the analysis that included gap characters only resulted in one more resolved node with no additional branches with bootstrap support >50%.

In addition to increasing the resolution and the number of branches with bootstrap support over 50%, the addition of gaps to the unweighted analysis resulted in higher overall bootstrap values. When we compared all the values for branches with bootstrap support >50% in the MP tree without gaps to those branches in the MP tree with gaps included, we found a net increase of 148 in the sum of bootstrap support values in the tree with gaps included. A similar comparison of bootstrap supported branches in the unweighted MP tree without gaps to the MP tree without gaps that incorporated the weighting of transversions to 1.5, resulted in only a net increase of 7 in the sum of bootstrap support values. Interestingly, when we compare bootstrap values in the unweighted MP tree without gaps to the MP tree with gaps added and both gaps and transversions given a weight of 1.5 (our most resolved tree), we see a net increase in the sum of bootstrap support values of only 132, or 16 less than when gaps were included with no weighting. While the weighting of transversions did provide some net increase in the sum of bootstrap support values on its own, when combined with the gap data, the weighting of gaps and transversion actually resulted in a lower sum of bootstrap support values than when gaps were added without any weighting. For our data set, the addition of coded gap characters to the phylogenetic analysis is more effective than the weighting of transversions in increasing both resolution and overall bootstrap support. These results, coupled with the congruence of the sequence data and the coded gap data, validate, and in fact encourage, the employment of sequence sets containing gaps in phylogenetic analysis. The value of gaps may derive from the fact that large indels, if their positions are very clearly definable in an alignment, may be less likely than base substitutions to be homoplastic.

We have labeled the two major allele clades defined by our phylogenetic analysis in a manner that provides consistency with prior studies. Our clade A contains all the sampled *F. vesca* alleles, as did the clade A defined by Potter et al. [3]. Also, in their genome composition models, Senanayake and Bringhurst [20] and Bringhurst [22] assigned the genome composition AA to *F. vesca*. Our clade B1 encompasses the five Asian species comprising Potter’s clade B (*F*. *nipponica*, *F*. *gracilis*, *F*. *pentaphylla*, *F*. *daltoniana*, and *F*. *nilgerrensis*), adding support to this grouping, but also includes three Asian species (*F*. *tibetica*, *F*. *corymbosa*, and *F*. *moupinensis*) that were not studied by Potter et al. [3]. Moreover, our clade B corresponds in membership to the clade X of Asian diploids and tetraploids delineated by Rousseau-Gueutin et al. [4], albeit in the latter study *F. yezoensis* (since folded into *F. nipponica* by Staudt and Olbricht [64]) was treated as distinct from *F. nipponica*.

The observed patterns of clustering of polyploid-derived alleles with diploid-derived alleles provides support for prior, botanically based phylogenetic hypotheses [10], [13], [14], as well as insights into the diploid sources of the respective polyploids’ *ADH-*1 alleles. Among the tetraploids, alleles of *F. orientalis* clustered in subclade A1 with those of *F. mandshurica*, its putative diploid ancestor [13]. While their putative relationships are not quite as clearly delineated, the alleles of *F. tibetica* and *F. moupinensis* clustered in subclade B2 with those of their putative diploid progenitors, *F. pentaphylla* and *F. nubicola* [10], [14] and other Asian diploids.

Our single representative of hexaploid *F. moschata* contributed three alleles to two distinct subclades: two alleles clustered with *F. viridis* alleles in subclade B2.2, implicating this diploid as a possible ancestral allele donor; and one allele resided in subclade A1, perhaps contributed by *F. vesca* or *F. mandshurica*, if not the sister subclade (A2) member *F. bucharica*. Thus, *F. moschata* likely has at least a partially allopolyploid genome composition, as first suggested by the AAAABB genome composition model of Fedorova [21], by whom this species was referred to as *F. elatior*. Examination of chloroplast DNA markers has provided evidence favoring *F. viridis* over *F. vesca*, *F. bucharica*, and *F. mandshurica* [47], [65] as the likely diploid source of the *F. moschata* chloroplast genome.

All octoploid-derived alleles fell into subclade A1 or subclade B2.1, and each octoploid possessed at least one allele belonging to each of these two divergent subclades. Thus, unlike the nuclear ITS and cpDNA based phylogenies of Potter et al. [3], which placed all octoploid-derived alleles in one clade, our results provide molecular documentation of the reticulate ancestries in the octoploid *Fragaria*. This differing outcome is not at all surprising. The octoploid *Fragaria* have long been viewed as allo- or alloauto- polyploids [20], [21], [22]. Moreover, a reticulate phylogeny could not be revealed by uniparentally transmitted cpDNA, and was not revealed by a nuclear ITS phylogeny [3], wherein genomic footprints may have been “smudged” by the homogenizing effects of gene conversion, converging on a single ITS allele type, and/or by loss of rDNA loci.

The distribution of all octoploid-derived alleles into just two distinct clades supports octoploid genome composition models that postulate two, but not three, distinct subgenome types [66]. Thus, our results are consistent with Model II - AAA’A’BBBB [20], Model III - AAA’A’BBB’B’ [22], and the two models proposed by Rousseau-Gueutin et al. [4], but not with Model I - AAAABBCC [21]. However, our results provide no insight into the postulated differentiation of A versus A’ and B versus B’ subgenomes.

In its allele composition, decaploid [39] accession PI641091 of the rather mysterious species *F. iturupensis* did not stand out in any noteworthy way from those of octoploids *F. chiloensis* and *F. virginiana*. The known geographic distribution of *F. iturupensis* is very narrow, being restricted to only a poorly accessible volcanic slope on the small island of Iturup, just north of Japan [38], [67]. In its original description *F. iturupensis* was characterized as octoploid [38], but the subsequent unavailability of plant samples precluded confirmation of ploidy. New accessions collected from Iturup in 2003 [67] were found to be decaploid [39].

The clustering of *F. vesca* and octoploid-derived alleles in clade A1 is consistent with the prior findings of Potter et al. [3] in supporting the widely held view that *F. vesca* is a likely ancestral genome contributor to the *Fragaria* octoploids [4], [9], [68]. Our findings also coincide with those of Rousseau-Gueutin et al. [4] in drawing attention to *F. mandshurica* and *F. iinumae* as potential allele donors, and perhaps genome donors, to the octoploids. As a member of clade A1 and a tetraploid derivative of *F. mandshurica* [13], *F. orientalis* is implicated as a potential conduit for allele and genome transfer from *F. mandshurica* to the octoploids.

Importantly, the clustering of some octoploid alleles with those of diploid *F. iinumae* implicates the latter species as a likely allele contributor to the octoploids, and perhaps a genome contributor that is highly genetically distinct from *F. vesca*. Based upon presentation of our preliminary results in scientific meetings [69], [70], *F. iinumae* has begun to garner considerable research attention [9], prompting the collection of a greatly expanded sampling of *F. iinumae* germplasm from Hokkaido, Japan [71], [72]. *F. iinumae* has often been cited as potentially ancestral to the octoploids on the basis of phenotypic resemblances [7], [11], [73], although until recently molecular confirmation was lacking. Interestingly, a hint of this close relationship was evident in a study of SSR (simple sequence repeat) primer pair transferability from octoploid to five diploid *Fragaria* species [51]. In that study, SSR primer pairs developed from *F*. ×*ananassa* sequence had the best amplification success rate (98.4%) in *F. vesca*, followed closely by *F. iinumae and F. bucharica* (both 93.8%) and more distantly by *F. nilgerrensis* (75%), and *F. viridis* (73.4%). Moreover, one *F*. ×*ananassa* -derived SRR primer pair, ARSFL_28, reproducibly amplified a product in only one diploid: *F. iinumae*.

The potential significance of *F. bucharica* in phylogenetic and genomic studies of *Fragaria* warrants careful consideration. *F. bucharica* was used as the parental crossing partner in the development of the FV×FB linkage map [50], which in turn anchored the *F. vesca* genome sequence [74]. Thus, the extent of its divergence from *F. vesca* has important implications for strawberry genomics. Moreover, as detailed in the Introduction, there has been a history of confusion in respect to the proper identification of *F. bucharica* germplasm accessions, suggesting a cautious approach in its consideration.

In our analysis of three *F. bucharica* accessions, the four distinguished alleles formed an exclusive subclade (A2) that was sister to the pivotal subclade (A1) containing *F. vesca*, *F. mandshurica*, and a bisect of the octoploid-derived alleles. Thus, although peripherally placed, *F. bucharica* resided with diploids *F. vesca* and *F. mandshurica* in strongly supported Clade A. As noted in the SSR study [51] cited above, *F. bucharica* ranked as high as *F. iinumae* and higher than *F. mandshurica* in success of marker transfer from *F*. ×*ananassa.* Rousseau-Gueutin et al. [4] studied a single *F. bucharica* accession, and found its positioning to be problematic and possibly indicative of hybridity. All evidence considered, we think it premature to draw any firm conclusions about the potential ancestral role of *F. bucharica*, and encourage an expansion of the germplasm and genomic resources available for study in this species.

In overview, the diploid species that were implicated as *ADH-*1 allele donors to the octoploids were (in clade A) *F. vesca*, *F. mandshurica*, and possibly *F. bucharica*, and (in clade B2.1) *F. iinumae*, while one or more clade A diploids and *F. viridis* (clade B2.2) are implicated as allele donors to hexaploid *F. moschata*. In contrast, the Asian species in clade B1 were not evident allele donors to the hexaploid and octoploid species. Conspicuously absent from the developing picture of octoploid ancestry are allopolyploids (tetraploids and/or hexaploids) possessing both clade A and clade B2.1 alleles, which might have served as evolutionary intermediates between the ancestral diploids and the octoploids. No octoploid-derived alleles fell into clade B2.2 with *F. viridis* and *F. moschata* alleles, countering the hypotheses that *F. moschata* might have been such an intermediate, or that *F. viridis* is an ancestor to the octoploids. In the absence of strong candidate species as evolutionary intermediates, a complete understanding of the octoploids’ ancestry may await identification of as yet undiscovered *Fragaria* species, providing a strong impetus to further germplasm exploration, collection, and evaluation efforts. This view is reinforced by the very recent discovery of decaploid *F. cascadensis*, a new *Fragaria* species that has been “hiding in plain sight” in the Cascade Mountains of Oregon [19]. Alternately, important *Fragaria* “missing links” may be no longer extant.

Additional insights into diploid-to-octoploid lineages in *Fragaria* have come from recent studies of chloroplast (cpDNA) and mitochondrial (mtDNA) markers and their modes of hereditary transmission. Although often considered by default to be maternally inherited, many examples of biparental or paternal transmission of organelle genomes exist in plants. Recently, the first molecular marker data demonstrating the maternal transmission of cpDNA [5] and mtDNA [6] in *Fragaria* have been presented. Intriguingly, although the cpDNA marker data agreed with the prior findings of Potter et al. [3] in implicating *F. vesca* as the cpDNA donor to the octoploids, *F. iinumae* was implicated by the limited available marker data as the source of the octoploids’ mtDNA. Pending confirmation of the latter finding by additional mtDNA marker or sequence data, it is hypothesized that exceptions to the generally observed, maternal pattern of organelle transmission may exist in *Fragaria*.

In summary, the highly polymorphic, intron-containing region of the *ADH-*1 gene proved to be a highly informative site for phylogenetic analysis. Additionally, the coherency of our analyses with and without inclusion of gaps as characters validates our focus on intron sequence, which is much more likely than exon sequence to be variable and to contain gaps. Our findings concerning the possible ancestry of the octoploid strawberry species have important implications for future directions in strawberry genomic research. In part because of its presumed ancestral status, but also because of its fecundity, self-fertility, ease of genetic transformation, diversity of mutant forms, and other favorable features, *F. vesca* has been justified and developed as a model system for strawberry genomics [9], and its genome has been sequenced to provide the first *Fragaria* reference genome [74]. While validating the attention given to *F. vesca*, our results point to the need for research investment in other diploids as well. As self-incompatible species, *F. mandshurica* and *F. bucharica* are distinct from self-compatible *F. vesca* and *F. iinumae*. Also, *F. iinumae* stands out from the aforementioned species by virtue of its strikingly glaucous leaves, thus resembling *F. virginiana* subsp. *glauca* [73], and by its acute sensitivity to powdery mildew (T.M. Davis, unpublished observations). Thus, each of these possibly ancestral diploids could be the source of unique alleles of relevance to a variety of agricultural traits, thereby providing valuable and relevant diploid systems within which to study these traits.

## Supporting Information

Table S1 **Germplasm accessions used in the phylogenetic study.** 1. Accessions with CFRA prefixes were obtained from the USDA National Clonal Germplasm Repository (NCGR) in Corvallis, Oregon. *F. vesca* cultivar ‘Yellow Wonder’ was initially purchased as seed from W. Atlee Burpee and Company, Warminster, Pennsylvania, and was subsequently seed propagated through natural self-pollination at the University of New Hampshire. SIB3 was obtained as seed from Garrett Crow, University of New Hampshire. Accessions U2A and BC21 were collected from the wild as runner plants by T. Davis. Leaf samples for DNA extractions from all accessions with GS prefixes were obtained from Günter Staudt, Merzhausen, Germany. 2. The accessions listed as *F. vesca* subsp. *bracteata* were collected within the range of this subspecies, as delineated by Staudt (1999), but have not been definitively typed to subspecies.
(XLSX)

Table S2 **Sequences used in phylogenetic analyses.** Allele lengths (in bp) are the sum of the number of bases in the listed sequence plus the number of bases (23 plus 23, respectively) in the forward and reverse primer sites, the sequences of which are not included in the listed allele sequences. Note: the table formatting is delimited by spaces and tabs. The data can be sorted by any column in Excel. The current listing order is defined in column CX and approximates the top-to-bottom order of allele appearance in the phylogenetic trees in Figure 3.
(XLSX)
